# Clasificación del nivel y grado de calcificación de canales radiculares mediante tomografía computarizada de haz cónico

**DOI:** 10.21142/2523-2754-1204-2024-219

**Published:** 2024-11-23

**Authors:** María Eugenia Terán-Miranda

**Affiliations:** 1 Centro Radiológico RD-MAX. Bogotá, Colombia. mariaeugeniateranrx@gmail.com Centro Radiológico RD-MAX Bogotá Colombia mariaeugeniateranrx@gmail.com

**Keywords:** cavidad pulpar, calcificación de la pulpa dental, endodoncia, tomografía computarizada de haz cónico (DeCS), dental pulp cavity, dental pulp calcification, endodontics, cone-beam computed tomography (DeCS)

## Abstract

**Objetivo:**

Evaluar la frecuencia de calcificación de canales radiculares (CCR) y proponer una clasificación del nivel y grado de calcificación de los CCR mediante tomografía computarizada de haz cónico (TCHC).

**Métodos:**

La muestra estuvo constituida por volúmenes tomográficos de 82 pacientes de ambos sexos (género femenino n = 61, 74,4%; masculino n = 21, 25,4%), con edades entre 41-71 años, de los cuales se analizaron 109 CCR. Se registró la localización del diente en el maxilar, el tipo de diente afectado, el tipo de canal calcificado, el nivel y el grado de calcificación; para estos últimos se diseñó una clasificación. El 30% de la muestra fue reevaluada por tres observadores independientes para validar las clasificaciones propuestas, y se obtuvo para ello una curva ROC.

**Resultados:**

La mayor frecuencia de CCR se encontró en el grupo de 40-49 años (23,85%), en el maxilar (n = 77, 70,64%) y el segundo cuadrante (44/109: 40,4%). Los dientes monorradiculares (43/109: 39,0%) y con único canal (51/109: 46%) fueron los más afectados. La calcificación cervical-medio-apical (31/109: 28,4%) y el grado de calcificación 3 (cerrado) (73/109: 67%) tuvieron las frecuencias más altas. Estos resultados mostraron un nivel de significancia de p < 0,05. La correlación para los evaluadores en la curva ROC fue, en promedio, de 0,89, lo que demuestra dominio en la observación de las variables.

**Conclusiones:**

La CCR se encontró con mayor frecuencia en dientes superiores monorradiculares y con único canal radicular, en individuos entre los 40 y 49 años edad. La clasificación propuesta puede constituirse en una guía visual para determinar el nivel y grado de la CCR utilizando la TCHC.

## Abstract (en)

**Objective:**

To evaluate the frequency of root canal calcification (RCC) and to propose a classification of the level and degree of RCC calcification by cone beam computed tomography (CBCT).

**Methods:**

The sample consisted of tomographic volumes of 82 patients of both sexes (female n=61, 74.4%; male n=21, 25.4%), aged 41-71 years, of which 109 RCC were analyzed. The location of the tooth in the maxilla, type of tooth affected, type of calcified canal, level and degree of calcification were recorded; a classification was designed for the latter. Thirty percent of the sample was reevaluated by three independent observers to validate the proposed classifications, obtaining a ROC curve.

**Results:**

The highest frequency of ROC was found in the 40-49 years age group (23.85%), in the maxilla (n= 77, 70.64%) and second quadrant (44/109-40.4%). Monoradicular (43/109-39.0%) and single canal (51/109-46%) teeth were the most affected. Cervical-mid-apical calcification (31/109-28.4%) and calcification grade 3 (closed) (73/109-67%) had the highest frequencies. These results showed a significance level of p<0.05. The correlation for the evaluators in the ROC curve was on average 0.89, demonstrating dominance in the observation of the variables.

**Conclusions:**

RCC was found more frequently in monoradicular and single root canal upper teeth, in individuals between 40 and 49 years of age. The proposed classification can be used as a visual guide to determine the level and degree of RCC through CBCT.

## INTRODUCCIÓN

El objetivo principal del tratamiento endodóntico (TE) es eliminar los irritantes del canal radicular (CR) y, una vez limpio y moldeado, rellenar adecuadamente el sistema de canales radiculares (SCR) para evitar cualquier recontaminación adicional ^1^. En este contexto, pueden ocurrir problemas que ocasionen el fracaso del TE por errores de procedimiento, y las causas más comunes son la no localización del CR y la instrumentación inadecuada ^2^.

Por tal motivo, el clínico debe estar familiarizado con la trayectoria que siguen los CR hasta llegar al ápice ^2^. Essan *et al*. ^3^ identificaron un total de 19 categorías asociadas con el riesgo de encontrar complejidad u obtener resultados adversos en el TE. Entre estos factores mencionan la calcificación pulpar, que se puede producir por el depósito de dentina secundaria a terciaria en la cavidad pulpar, debido a procesos patológicos como caries, así como restauraciones o cambios relacionados con la edad del paciente. Se manifiesta a lo largo de las paredes de la cámara y el CR, o como la formación de puentes de dentina en el orificio del CR ^4^ De igual manera, puede causar disminución del espacio pulpar la presencia de cálculos ^5^ o la denominada calcificación distrófica, identificada como un depósito rápido de tejido duro producto de una reacción pulpar al traumatismo ^6^. Estos procesos pueden conducir a la obliteración parcial o completa de la pulpa cameral y el espacio del CR, e incluso la obstrucción apical, lo que aumenta el riesgo de errores de procedimiento durante la preparación del CR ^3^^,^^6^^,^^7^. 

En los casos de calcificación del CR, el tiempo del tratamiento suele prolongarse y es laborioso, lo que resulta en menor cooperación del paciente y una mayor tasa de fracaso ^8^. La Asociación Americana de Endodoncia (AAE) ^9^ considera que el manejo de la CCR es un procedimiento con alto nivel de dificultad y complejidad, tanto por la ubicación de los orificios del CR como por la preparación biomecánica. Además, su presencia suele estar asociada con un desgaste excesivo de la estructura dental, además de un mayor riesgo de desviación del canal y perforación radicular. El grado de dificultad está dictado por la morfología del diente, la naturaleza y extensión de la calcificación, la extensión de la esclerosis dentinaria y el acceso al diente en boca ^10^. 

En el abordaje de la TE, la recopilación de información pertinente es prioritaria para el establecimiento de un diagnóstico apropiado y preciso. En este contexto, las radiografías preoperatorias son una parte indispensable de los procedimientos de diagnóstico; sin embargo, la valoración de casos complejos con radiografías convencionales puede ser un desafío ^11^. En la actualidad, la tomografía computarizada de haz cónico (TCHC) ha permitido una mejor comprensión anatómica del SCR y las patologías asociadas, gracias a su visualización en los diferentes planos del espacio ^11^^-^^13^. 

La AAE y la Asociación Americana de Radiología Oral y Maxilofacial, en su documento de consenso, recomiendan la obtención del volumen de TCHC empleando un campo de visión limitado (FOV, por sus siglas en inglés) de 40 a 50 mm de diámetro, que puede limitarse a varios dientes ^11^^-^^14^, siendo la modalidad de elección para el diagnóstico del paciente que presente signos clínicos o no específicos de síntomas asociados con dientes tratados endodónticamente o no ^6^. La TCHC proporciona valiosas mediciones de profundidad para la detección del CR, lo que facilita un acceso predecible durante el tratamiento ^6^.

A fin de obtener un acceso adecuado a la CCR, se han utilizado distintas estrategias como el uso de microscopio dental, lupas, puntas ultrasónicas y agentes quelantes. Actualmente, se ha desarrollado un enfoque asistido por computador mediante el cual se obtienen “guías endodónticas” al sobreponer los archivos DICOM (*Digital Imaging and Communications in Medicine*) obtenidos de un estudio tomográfico, con los archivos STL (*Standard Tesselation Language*) adquiridos mediante un escáner ^15^^-^^18^. La guía permite ganar acceso a la parte permeable del CR con una fresa de diámetro que varía entre 0,75 a 1,2 mm, lo que minimiza riesgos procedimentales ^18^^,^^19^.

El enfoque terapéutico para tratar una CCR es variado. Algunos autores aún recomiendan el TE preventivo ante la presencia de signos de reparación pulpar, o incluso cuando solo se sospecha la aparición de dichos procesos ^20^. Sin embargo, estos tratamientos pueden provocar errores iatrogénicos tales como perforaciones o fracturas de instrumentos, entre otros ^21^. En este sentido, Fonseca Tavares *et al*. ^22^ eligieron la técnica de endodoncia guiada como el tratamiento más apropiado después de intentos fallidos de localizar el CR en un caso con periodontitis apical. Lakinepally *et al*. ^23^, en el caso de obliteración parcial del CR con diagnóstico de periodontitis apical sintomática, realizaron un TE no quirúrgico. Por tal motivo, caracterizar el CR para ubicar la calcificación u obliteración es una tarea primordial, y la evaluación con TCHC permite esas precisiones terapéuticas ^21^.

El concepto de endodoncia mínimamente invasiva también ha creado la necesidad de comprender mejor la anatomía de la cámara pulpar y el CR ^24^. Desde esa perspectiva, es una condición básica para la eficiencia endodóntica que el clínico posea una visión holística de la morfología del canal radicular del diente a tratar ^2^ y, en consecuencia, tener a disposición indicaciones precisas que le permitan realizar el procedimiento endodóntico que preserve la mayor cantidad de tejido dentario, lo cual es una fortaleza para su ejercicio. Asimismo, a través de las imágenes provistas por la TCHC, puede abordar satisfactoriamente la CCR y alcanzar la porción permeable. Estas consideraciones orientan el objetivo de la presente investigación sobre una propuesta de clasificación del nivel y grado de calcificación de los canales radiculares mediante TCHC, lo que le permite al clínico un desempeño exitoso en el abordaje para el TE.

## MATERIALES Y MÉTODOS

Se realizó una investigación descriptiva, transversal y retrospectiva. La muestra estuvo constituida por 82 volúmenes de TCHC seleccionados de forma intencional, pertenecientes a pacientes de ambos sexos (61-74: 4% de sexo femenino y 21-25: 6% de sexo masculino) referidos a un centro radiológico privado, con edades entre 41 y 71 años (media: 56 ± 15 años). Para la selección de la muestra se consideraron los siguientes criterios de inclusión: a) Examen tomográfico indicado con fines endodónticos por sospecha clínica o radiográfica de dientes con CCR; b) Examen tomográfico con FOV reducido y de alta resolución; c) Volumen tomográfico sin artefactos de imagen generados por objetos metálicos o movimiento que imposibilitaran la evaluación de las estructuras de interés. Todos los exámenes de TCHC fueron obtenidos por razones clínicas, de manera que no hubo una exposición adicional del sujeto a la radiación ionizante, de acuerdo con las directrices de la Declaración de Helsinski ^25^ para el estudio en seres humanos.

### Adquisición del volumen tomográfico

Las imágenes fueron adquiridas en un equipo de TCHC Promax 3D (Planmeca; Helsinki, Finlandia), con 90 kVp, 10 mA en media y un tiempo de exposición promedio de 14 segundos, FOV de 5,0 x 5,0 mm y un vóxel de 0,75 mm. El kVp y el mA pudieron ser ajustados de acuerdo a la contextura y tamaño del paciente. Se utilizaron los filtros reducción de artefactos (ARA, Planmeca; Helsinki, Finlandia) y reducción de movimiento (CALM, Planmeca; Helsinki, Finlandia). Todas las imágenes fueron almacenadas en formato DICOM (*Digital Imaging and Communication in Medicine*). 

### Análisis del volumen tomográfico

La evaluación de las imágenes tomográficas fue realizada utilizando el *software* Romexis 6.0 (Planmeca; Helsinki, Finlandia) por un observador. Se utilizó una estrategia de mapeamiento dinámico del volumen tomográfico ^26^^,^^27^, la cual consiste principalmente en la localización de la raíz en estudio en la ventana de exploración multiplanar del *software* para ajustar los ejes axial, coronal y sagital, en la misma dirección del eje mayor de la raíz, y después realizar un recorrido en dirección corono-radicular y viceversa, empleando la menor distancia entre cortes, al menor espesor posible. 

A continuación se registró el tipo de diente afectado (monorradicular, birradicular y multirradicular) y el tipo de canal radicular afectado, que puede ser único (U): el diente presenta un solo canal radicular; vestibular (V): canal radicular localizado en la raíz vestibular; palatino (P): canal radicular localizado en la raíz palatina; mesiovestibular (MV): canal radicular localizado hacia vestibular de la raíz mesial del molar; mesiovestibular 2 (MV2): canal radicular localizado hacia palatino de la raíz mesiovestibular de un molar superior; mesiolingual (ML): canal radicular localizado hacia lingual en la raíz mesial del molar inferior; distal (D): canal radicular localizado en la raíz distal del molar inferior; distovestibular (DV): canal radicular localizado hacia vestibular de la raíz distal de un molar superior. 

El nivel de la calcificación, definido como la zona del canal radicular donde se ubicó tomográficamente la calcificación en los tres planos del espacio, fue determinado de acuerdo con la siguiente clasificación:

• Pulpar (P): la calcificación se localiza únicamente en la cámara pulpar.
• Cervical (C): la calcificación se localiza únicamente en el tercio cervical radicular.
• Medio (M): la calcificación se localiza únicamente en el tercio medio radicular.
• Apical (A): la calcificación se localiza únicamente en el tercio apical radicular.
•Pulpar-cervical (PC): la calcificación involucra la cámara pulpar y el tercio cervical radicular.
• Cervical-medio (CM): la calcificación involucra los tercios cervical y medio radicular.
• Medio-apical (MA): la calcificación involucra los tercios medio y apical radicular.
• Pulpar-cervical-medio (PCM): la calcificación involucra la cámara pulpar y los tercios cervical y medio radicular.
• Cervical-medio-apical (CMA): la calcificación involucra los tercios cervical, medio y apical radicular.
• Total (T): la calcificación involucra cámara pulpar y los tres tercios del canal radicular.

El grado de calcificación del canal radicular fue clasificado en:

• Grado 1-Obliterado: describe un lumen radicular adelgazado o muy fino con relación a los canales radiculares permeables de otros dientes homólogos del mismo paciente.
• Grado 2-Intermitente: describe un lumen del canal radicular con áreas visibles y no visibles.
• Grado 3-Cerrado: describe la no visualización del lumen del canal radicular.

**Figura 1:**
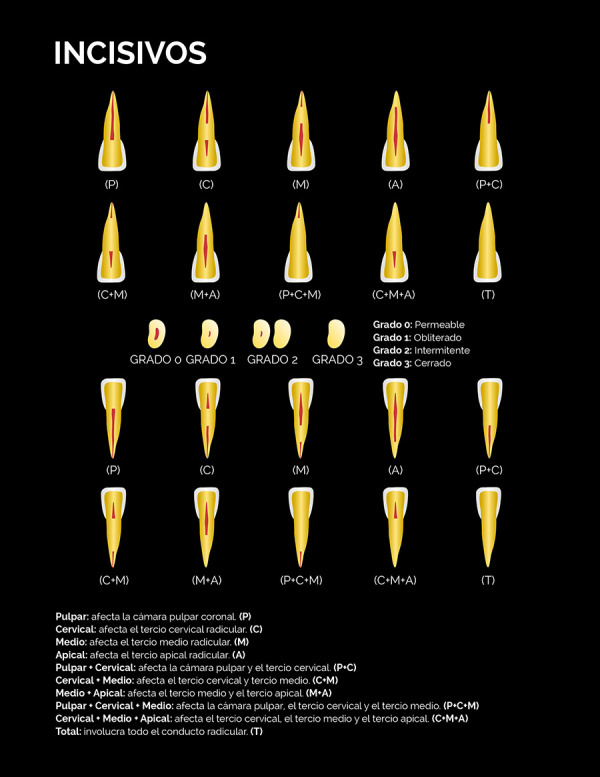
Representación esquemática de la clasificación del nivel y grado de calcificación del canal radicular mediante tomografía computarizada de haz cónico para los incisivos.

Estas clasificaciones están representadas gráficamente en las figuras 1 al 4. Con la finalidad de validar la clasificación del nivel y grado de calcificación, tres observadores fueron calibrados mediante la observación de casos de CCR no incluidos en la muestra siguiendo un instructivo escrito y dirimiendo las discrepancias en consenso. Posteriormente, evaluaron de forma independiente un 30% de los volúmenes tomográficos de la muestra seleccionados de forma aleatoria simple. 

**Figura 2:**
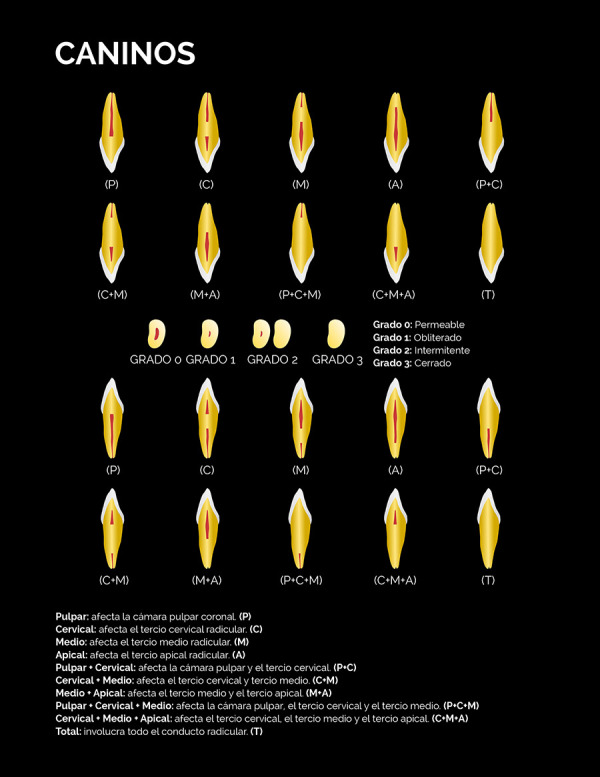
Representación esquemática de la clasificación del nivel y grado de calcificación del canal radicular mediante tomografía computarizada de haz cónico para los caninos.

**Figura 3:**
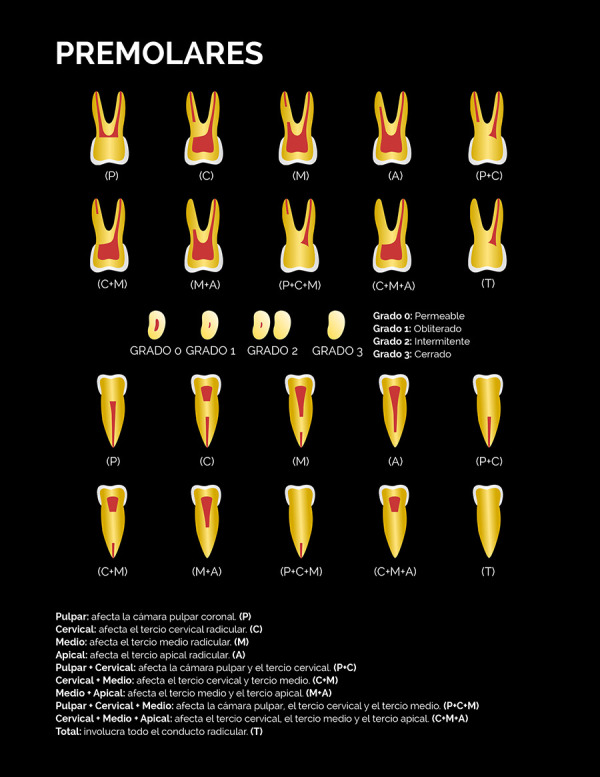
Representación esquemática de la clasificación del nivel y grado de calcificación del canal radicular mediante tomografía computarizada de haz cónico para los premolares.

**Figura 4:**
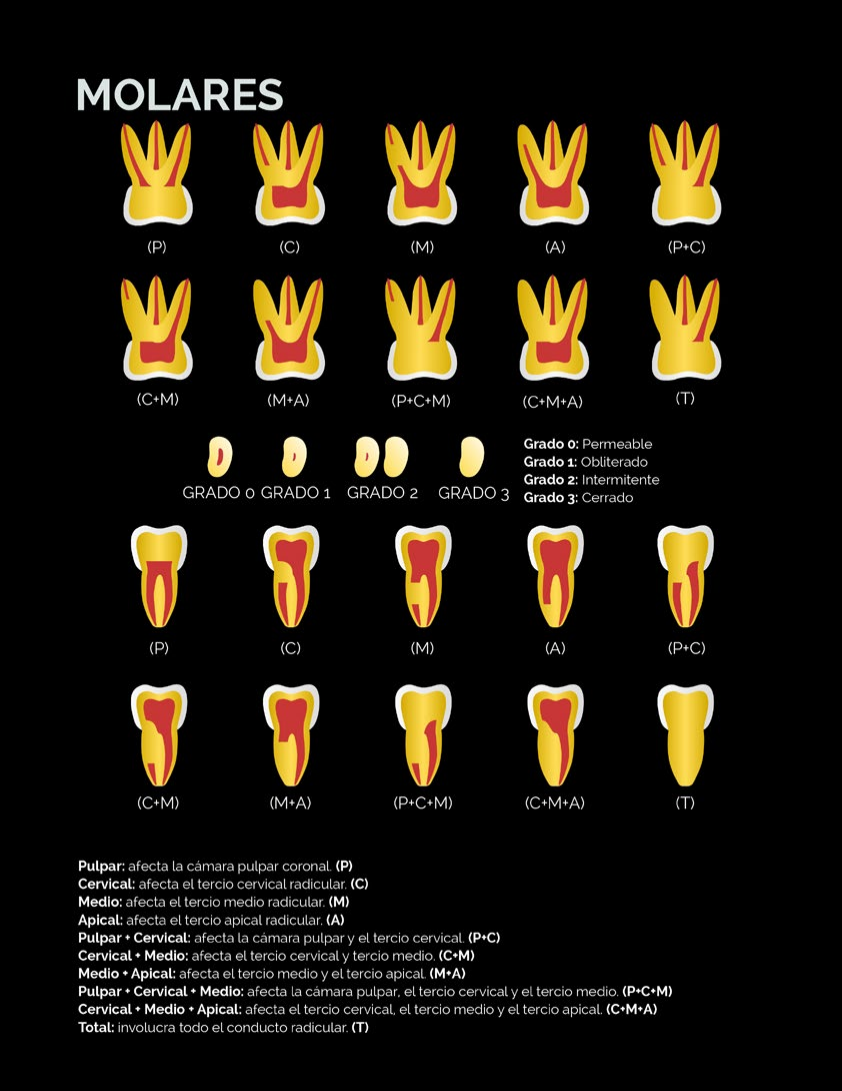
Representación esquemática de la clasificación del nivel y grado de calcificación del canal radicular mediante tomografía computarizada de haz cónico para los molares.

### Análisis de los datos

El análisis estadístico de los datos se realizó mediante el paquete estadístico SPSS (IBM Corp. Released 2020. IBM SPSS Statistics for Windows, Version 27.0. Armonk, NY: IBM Corp.). Se obtuvieron frecuencias de la CCR de acuerdo al sexo y grupo de edad, tipo de diente afectado, nivel y grado de calcificación. Se aplicó el test de chi-cuadrado para evaluar la existencia de alguna asociación. Se empleó un análisis de curva ROC (*Receiver Operating Characteristic Curve*) para conocer la concordancia de los resultados obtenidos por los observadores al aplicar las clasificaciones propuestas. El nivel de significancia asumido fue de p < 0,05.

Las medidas de asociación o correlación para la curva ROC se interpretaron mediante los siguientes rangos (Spearman rho): a) Rho entre 0-0,25 existe una correlación escasa o nula; b) Rho entre 0,26-0,50 existe una correlación débil; c) Rho entre 0,51-0,75 existe una correlación moderada; d) Rho entre 0,76-1 existe una correlación fuerte o perfecto.

## RESULTADOS

Se evaluaron 109 raíces correspondiendo a 109 dientes, de estas 81 (74,3%) pertenecían al género femenino y 28 al género masculino (25,7%), en una proporción de 2,29:1, lo que fue estadísticamente significativo (p < 0,05). Se obtuvo la distribución de las raíces con CCR de acuerdo al rango de edad, y se encontró que el grupo de edad con mayor frecuencia de calcificación pulpar fue el comprendido entre 40 a 49 años, con 26/109 (23,85%), seguido por los grupos de 60 a 69 años, con 22/109 (20,18%), y el 30 a 39 años, con 20/109 raíces (18,35%). 

**Tabla 1:** Distribución de la calcificación de los canales radiculares calcificados de acuerdo al grupo de edad.

Rangos de edad	Frecuencia	Porcentaje
20-29	2	1,83%
30-39	20	18,35%
40-49	26	23,85%*
50-59	15	13,76%
60-69	22	20,18%
70-79	17	15,60%
Más de 80	7	6,42%
Total	109	100%

*Estadísticamente significativo a p < 0,05, de acuerdo con test de chi-cuadrado.

En relación con la distribución de CCR por tipo de diente se observó una mayor tendencia en los dientes monorradiculares, correspondiendo a 51/109 casos (46,8%), seguido de los de los dientes con tres raíces con 43/109 casos (39%), con un nivel de significancia de p < 0,05 y en menor frecuencia en los dientes con dos raíces en 15/109 casos (13,8%). Asimismo, se obtuvo la distribución del tipo de canales calcificados, encontrándose que los dientes que presentan un único canal radicular son los más frecuente en las 109 raíces estudiadas, siendo este de 51/109 (46%) con significancia estadística, sigue el DV (14/109: 12,8%), P (13/109: 11,93%), MV (11/109: 10%), MV2 (8/109: 7,34%), ML (5/109: 4,59%) y V (4/109: 3,68%). 

La calcificación cervical-medio-apical (31/109: 28,4%) y el grado de calcificación 3 (cerrado) (73/109: 67%) tuvieron las frecuencias más altas.

Cuando se investigó la distribución de las raíces con CCR según el grupo dentario, maxilar y cuadrante (tabla 2), se encontró que 77 (70,64%) de los canales radiculares calcificados estuvieron localizados en el maxilar y 32 (29,36%) en la mandíbula. Al considerar los cuadrantes, el cuadrante II mostró mayor frecuencia de canales radiculares calcificadas, con 44/109 raíces (40,4%), y el cuadrante I, con 33/109 raíces (38, 2%). Respecto de los cuadrantes III y IV, estos mostraron porcentajes semejantes. En el maxilar, los primeros molares, seguidos por los segundos molares y los incisivos centrales, presentaron los mayores porcentajes de CCR, lo cual fue estadísticamente significativo para los primeros y segundos molares. En la mandíbula, los incisivos centrales, primeros molares, incisivos laterales y caninos tuvieron los porcentajes más altos.

**Tabla 2:** Distribución de la calcificación de los canales radiculares calcificados de acuerdo con el grupo dentario y maxilar.

	**Grupo dentario**	I Cuadrante	II Cuadrante	Total	Porcentaje
Maxilar	Incisivos centrales	6	4	10	9,17%
	Incisivos laterales	3	4	7	6,42%
	Caninos	2	5	7	6,42%
	Primeros premolares	4	2	6	5,50%
	Segundos premolares	4	1	5	4,59%
	Primeros molares	8	20	28	25,69%*
	Segundos molares	6	8	14	12,84%
	Total	33	44	77	70,64%*
	Porcentaje	30,28%	40,37%	70,64%	
		III cuadrante	IV cuadrante	Total	Porcentaje
Mandíbula	Incisivos centrales	4	4	8	7,34%
	Incisivos laterales	3	2	5	4,59%
	Caninos	3	2	5	4,59%
	Primeros premolares	2	2	4	3,67%
	Segundos premolares	3	-	3	2,75%
	Primeros molares	1	6	7	6,42%
	Segundos molares	-	-	0	0,00%
	Total	16	16	32	29,36%

*Estadísticamente significativo a p < 0,05, de acuerdo con test de chi cuadrado.

Considerando el nivel de la calcificación, se puede observar en la tabla 3 que existió una significancia estadística para las calcificaciones de nivel CMA con un 28,4% representados por 31/109 raíces estudiadas, seguidas por calcificaciones de nivel A con 21/109 raíces estudiadas, con un 19,2%, y calcificaciones de nivel cervical con 16/109 raíces estudiadas, con un 14,7%, y calcificaciones de nivel PC, con 11/109 raíces estudiadas, con un 10%. El resto de las categorías mostraron una frecuencia menor al 10%. La tabla 4 presenta la distribución del grado de CCR y se evidenció una significancia estadística en las calcificaciones grado 3, con un 67% representados por 73/109 raíces estudiadas, seguidas por el grado 2 y grado 1. 

**Tabla 3:** Distribución del nivel de calcificación en la muestra.

Nivel de calcificación	Frecuencia	Porcentaje
Pulpar (P)	6	5,50%
Cervical (C)	16	14,68%
Medio (M)	3	2,75%
Apical (A)	21	19,27%
Pulpar-cervical (PC)	11	10,09%
Cervical-medio (CM)	3	2,75%
Medio-apical (MA)	9	8,26%
Pulpar-cervical-medio (PCM)	3	2,75%
Cervical-medio-apical (CMA)	31	28,44%*
Total (T)	6	5,50%
Total	109	100%

*Estadísticamente significativo a p < 0,05, de acuerdo con test de chi cuadrado.

**Tabla 4:** Distribución del nivel de calcificación en la muestra.

Grado de calcificación	Frecuencia	Porcentaje
Grado 1	18	16,51%
Grado 2	18	16,51%
Grado 3	73	66,97%*
Total	109	100%*

*Estadísticamente significativo a p < 0,05, de acuerdo con test de chi cuadrado.

**Figura 5:**
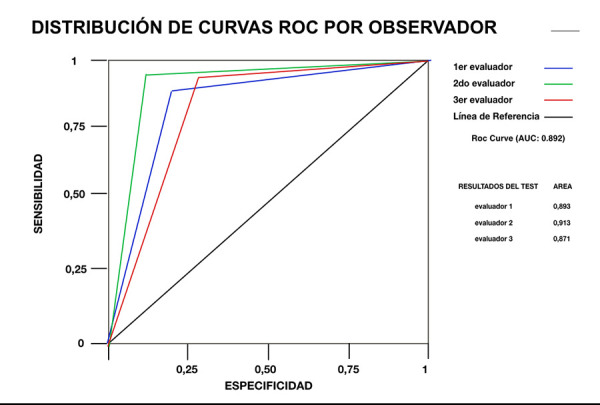
Distribución de curvas ROC (*Receiver Operating Characteristic Curve*) por observador en relación con las variables nivel y grado de calcificación del canal radicular.

Con respecto al análisis de curva ROC (Fig. 5), los valores obtenidos para los tres evaluadores, presentaron una correlación promedio de 0,89 (evaluador 1: 0,893; evaluador 2: 0,913; evaluador 3: 0,871), lo que evidenció dominio por parte de los participantes tanto en la observación como en la interpretación de las variables implicadas.

## DISCUSIÓN

En el complejo pulpo-dentinario se producen cambios progresivos debido a causas tanto fisiológicas como patológicas. Ciertamente, los canales calcificados presentan un desafío práctico para el clínico durante el TE, con un riesgo de perforación alto, y por ello se debe ser cauteloso al localizar e instrumentar los CR ^7^. La tasa de fracaso del TE en CCR oscila entre el 20% y el 70%. Este amplio rango de valores está influenciado por la experiencia clínica y el conocimiento anatómico del CR por parte del operador, así como por la información proporcionada por la radiografía bidimensional y las evaluaciones tridimensionales ^6^; de allí la importancia de contar con imágenes que puedan aportar información relevante. La TCHC de alta resolución y campo reducido permite la visualización del CR con mayor detalle y precisión, tal como se observó en esta investigación. 

La calcificación del espacio pulpar dental es un hallazgo clínico común. Se encuentra en dientes cariados, posterior al tratamiento ortodóntico e, incluso, en dientes maduros clínicamente sanos, principalmente en adultos mayores ^28^. En este estudio se evidenció un mayor porcentaje de CCR en pacientes de 40-49 años, seguidos por los individuos de 60-69 años. Johnstone y Parashos ^7^ concluyeron que, a medida que aumentaba la edad del paciente, se presentaba la calcificación bien sea en cámara pulpar o en los CR. Aunque este estudio coincide en la aseveración de que los adultos son los mayormente afectados con CCR, se expresó de manera diferente porque no fue progresiva con la edad, es decir, el porcentaje elevado correspondió a los pacientes de 40 a 49 años, disminuyó para la década de los 50 y aumentó en menor proporción en los sujetos de 60 años a más.

Con referencia a las piezas dentarias afectadas, los estudios plantean resultados diversos. Se informa que la obliteración del canal pulpar ocurre entre el 69% y el 73% de los incisivos afectados por fracturas radiculares ^6^. En el presente estudio se encontró una similitud respecto de la distribución de CCR por tipo de diente, ya que se observó una mayor tendencia de CCR en dientes monorradiculares.

En estudios sobre CCR, la terminología empleada con respecto al “grado de calcificación” se refiere a la misma como “total” o “localizada”. ^21^^,^^28^^,^^29^, mientras que en la presente investigación se incorpora el término “intermitente”, el cual describe una calcificación por segmentos que puede observarse tomográficamente en el CR calcificado. Con referencia al “nivel de la calcificación”, se consideraron los tercios radiculares cervical, medio y apical (C-M-A), términos utilizados en la investigación de Fornara *et al*. ^8^. Es importante destacar que, en la clasificación propuesta en este trabajo, se incorporan distintas categorías para identificar si se trata de un solo tercio afectado o varios, con la finalidad de que el clínico pueda precisar con mayor exactitud la ubicación y extensión que debe transitar para lograr la permeabilidad del CR.

Al evaluar el grado de la CCR, se evidenció una significancia estadística en las calcificaciones grado 3, donde no se visualiza el lumen del CR, es decir, está cerrado, y en cuanto al nivel, hubo una significancia estadística para las calcificaciones radiculares en cervical-medio-apical. Según lo desarrollado por Mac Cabe y Dummer ^30^, el proceso de obliteración radicular sucede en dirección corono-apical. En lo reportado por Fleig *et al*. ^28^, los procesos de obliteración o calcificación ocurren en la cámara pulpar, por un lado, y en el espacio pulpar radicular, por el otro. En dicha muestra, la tendencia fue el estrechamiento de CR desde coronal hacia apical. En este estudio, la CCR se ubicó en mayor proporción a nivel del tercio apical. En consideración el nivel de calcificación, la obliteración completa del espacio del CR es rara ^6^ a similitud de los resultados evidenciados en este estudio que representó un 3%. 

Aunque se han descrito técnicas diversas para tratar la CCR, incluso los endodoncistas más experimentados pueden tener dificultades para alcanzar la permeabilidad y realizar la limpieza y el modelado adecuados ^31^. Dependiendo del caso y del criterio del clínico, varía el abordaje y el tratamiento en los casos de CCR. Por tal motivo, el contar con una clasificación que permita identificar el nivel de la calcificación y el grado de la afectación del CR, puede orientar la estrategia más adecuada para el TE, con resultados más predecibles y perdurables en el tiempo, reduciendo errores procedimentales. 

Limitaciones:

La principal limitación de este estudio fue el tamaño de la muestra y el tipo de dientes estudiados. Al ampliarla, existiría una mayor probabilidad de seleccionar dientes con más de un canal calcificado y con una conformación del sistema de canales radiculares de mayor complejidad; esto posibilitaría evaluar la aplicabilidad de la clasificación en estos casos y realizar ajustes para dar respuesta a las distintas situaciones clínicas. 

## CONCLUSIÓN

En este estudio, la calcificación del canal radicular se encontró con mayor frecuencia en dientes superiores, monorradiculares y con único canal radicular, en individuos entre los 40 y 49 años edad. La calcificación cervical-medio-apical y el grado de calcificación 3 (cerrado) tuvieron las frecuencias más altas. El tratamiento endodóntico requiere en los casos de calcificación del canal radicular, el manejo de conocimientos sólidos sobre la conformación tridimensional del sistema de canales radiculares. El contar con una guía visual que indique el nivel y el grado de la calcificación en el canal radicular utilizando tomografía computarizada de haz cónico, puede constituir una herramienta útil para el diagnóstico y la planificación de la terapéutica por implementar.

## Fuentes de financiamiento:

Este trabajo no tuvo fuente de financiamiento
